# The Hospital Officers' Association

**Published:** 1904-05-21

**Authors:** J. Stephen Neil


### THE HOSPITAL OFFICERS' ASSOCIATION.

*To the Editor of* THE HOSPITAL.

SIR,—I have had my attention drawn to the obituary notice of Mr. Adrian Hope in which the writer expresses his belief that the late Mr. Hope was "parent" to the Hospital Officers' Association. This allusion is subject to varied meanings, one of which—the one leading the reader to regard Mr. Hope as founder of that Association—I trust, bears correction. The Hospital Officers' Association was founded and placed before the hospital public by myself; but this statement is made with all deference to the memory of a man I deeply admired—the late Mr. Adrian Hope. J. STEPHEN NEIL.

